# OLD-AGE DEPRESSIVE SYMPTOMS: THE ROLE OF EARLY-LIFE FINANCIAL STRAIN AND LATE-LIFE SOCIAL ENGAGEMENT

**DOI:** 10.1093/geroni/igz038.016

**Published:** 2019-11-08

**Authors:** Federico Triolo, Linnea Sjöberg, Davide Vetrano, Alexander Darin-Mattsson, Marco Bertolotti, Laura Fratiglioni, Serhiy Dekhtyar

**Affiliations:** 1 Aging Research Center, Department of Neurobiology, Care Sciences and Society, Karolinska Institutet and Stockholm University, Stockholm, Sweden; 2 Department of Biomedical, Metabolic and Neural Sciences, University of Modena and Reggio Emilia, Modena, Italy

## Abstract

It remains unclear if childhood socioeconomic disadvantage is associated with depression in old age. This study aims to investigate the effect of childhood financial strain on depressive symptoms in old age, and to examine whether late-life social engagement modifies this association. Data from the Swedish National study of Aging and Care in Kungsholmen, a community-based longitudinal study of aging, spanning clinical assessments over 15 years of follow-up were used. Information on financial strain in childhood was collected at baseline. Repeated measures of the Montgomery–Åsberg Depression Rating Scale were used to define depressive trajectories. A social engagement index comprised information on baseline social network and leisure activities. Linear mixed models were used to estimate depressive trajectories. Childhood financial strain was associated with a higher level of depressive symptoms (β = 0.36; p<0.05), but not the rate of symptom accumulation over time. Relative to those with a combination of no financial strain and active social engagement, the level of depressive symptoms was progressively increased in those without financial strain but with inactive social engagement (β = 0.29; p<0.05), as well as in those with both financial strain and inactive engagement (β= 0.83; p<0.05). Individuals with financial strain who had active social engagement exhibited a similar burden of symptoms as those without financial strain and with rich social engagement. Early-life financial strain may have a lasting effect on old age depressive symptoms, although its detrimental consequences may be modified by active social engagement in late life.

